# Repurposing celecoxib as a topical antimicrobial agent

**DOI:** 10.3389/fmicb.2015.00750

**Published:** 2015-07-28

**Authors:** Shankar Thangamani, Waleed Younis, Mohamed N. Seleem

**Affiliations:** Department of Comparative Pathobiology, Purdue University College of Veterinary Medicine, West Lafayette, INUSA

**Keywords:** celecoxib, antimicrobial resistance, repurposing, skin infection, anti-inflammatory

## Abstract

There is an urgent need for new antibiotics and alternative strategies to combat multidrug-resistant bacterial pathogens, which are a growing clinical issue. Repurposing existing approved drugs with known pharmacology and toxicology is an alternative strategy to accelerate antimicrobial research and development. In this study, we show that celecoxib, a marketed inhibitor of cyclooxygenase-2, exhibits broad-spectrum antimicrobial activity against Gram-positive pathogens from a variety of genera, including *Staphylococcus, Streptococcus, Listeria, Bacillus*, and *Mycobacterium*, but not against Gram-negative pathogens. However, celecoxib is active against all of the Gram-negative bacteria tested, including strains of, *Acinetobacter*, and *Pseudomonas*, when their intrinsic resistance is artificially compromised by outer membrane permeabilizing agents such as colistin. The effect of celecoxib on incorporation of radioactive precursors into macromolecules in *Staphylococcus aureus* was examined. The primary antimicrobial mechanism of action of celecoxib was the dose-dependent inhibition of RNA, DNA, and protein synthesis. Further, we demonstrate the *in vivo* efficacy of celecoxib in a methicillin-resistant *S. aureus* (MRSA) infected *Caenorhabditis elegans* whole animal model. Topical application of celecoxib (1 and 2%) significantly reduced the mean bacterial count in a mouse model of MRSA skin infection. Further, celecoxib decreased the levels of all inflammatory cytokines tested, including tumor necrosis factor-α, interleukin-6, interleukin-1 beta, and monocyte chemo attractant protein-1 in wounds caused by MRSA infection. Celecoxib also exhibited synergy with many conventional antimicrobials when tested against four clinical isolates of *S. aureus*. Collectively, these results demonstrate that celecoxib alone, or in combination with traditional antimicrobials, has a potential to use as a topical drug for the treatment of bacterial skin infections.

## Introduction

Bacterial infections caused by multi-resistant pathogens have emerged as a major global crisis during the past few decades (Fischbach and Walsh, 2009). The U.S. Centers for Disease Control and Prevention [CDC] (2013) indicated that at least two million individuals per year in the U. S. becomes infected with multidrug-resistant pathogens, including methicillin-resistant *Staphylococcus aureus* (MRSA), and multidrug-resistant *Pseudomonas aeruginosa*. More importantly, the emergence and spread of multidrug-resistant *S. aureus* clones such as MRSA USA100, USA200, and USA300 are approaching epidemic proportions and becoming a major global health concern (Diep et al., 2006; King et al., 2006; Seybold et al., 2006; Davis et al., 2007; Tenover et al., 2008, Tattevin et al., 2009; Tenover and Goering, 2009; Tong et al., 2009; Stryjewski and Corey, 2014). Clones like USA300 are highly virulent and cause skin and soft tissue infections that lead to morbidity and mortality in infected patients (King et al., 2006). Furthermore, the exo-proteins and toxins secreted by these MRSA strains trigger excess host inflammatory responses and further complicate the situation, especially in the management of wound infections (Fournier and Philpott, 2005; Diep et al., 2006, 2012; Gordon and Lowy, 2008). Further complicating the problem, there is increasing incidence of staphylococcal resistance to topical antimicrobials such as mupirocin and fusidic acid (Dobie and Gray, 2004; Kresken et al., 2004; Chambers and Deleo, 2009). Although there are several new approved systemic antibiotics available to treat skin infections such as oritavancin, tedizolid, there is unmet need for novel topical antimicrobial capable of modulating the host immune response and reducing the excessive inflammation associated with bacterial skin infections without exposing the patient to a systemic antibacterial agent.

The development of new antimicrobials is very slow process and has not been able to keep pace with the emergence of bacterial resistance (Fischbach and Walsh, 2009). Hence, novel drugs and treatment strategies are urgently needed to combat these bacterial pathogens. Repurposing of approved drugs is a promising alternative strategy that can accelerate the process of antimicrobial research and development (Rangel-Vega et al., 2015; Thangamani et al., 2015). Unlike conventional drug discovery, finding new uses for existing drugs is a proven shortcut from bench to bedside, that reduces the cost and time associated with antibiotic development (Ashburn and Thor, 2004; Chong and Sullivan, 2007; Rangel-Vega et al., 2015; Thangamani et al., 2015).

Celecoxib (Celebrex) is a non-steroidal anti-inflammatory drug widely used for the treatment of pain, fever, and inflammation (Frampton and Keating, 2007; McCormack, 2011). It specifically inhibits the enzyme cyclooxygenase-2 (COX2), thereby reducing the synthesis of proinflammatory prostaglandins (Bensen, 2000). Beyond its anti-inflammatory activity, celecoxib has been shown to possess antimicrobial activity against several microbial pathogens. In a study by Pereira et al. (2013) celecoxib was found to reduce the total fungal load in *Histoplasma capsulatum* infected mice. Further, celecoxib treatment also increased the survival rate of the mice infected with lethal dose of *H. capsulatum* (Pereira et al., 2013). Another study by Chiu et al. (2009) found that celecoxib inhibited the growth of *Francisella tularensis* and *F. novicida*. In addition, celecoxib also exhibited antibacterial activity against *S. aureus* and *S. epidermidis* (Chiu et al., 2012). Apart from antimicrobial activity, celecoxib inhibits multidrug efflux pumps in *Mycobacterium smegmatis* and *S. aureus*, and increases the sensitivity of bacteria to various antibiotics, including ampicillin, kanamycin, ciprofloxacin, and chloramphenicol (Kalle and Rizvi, 2011; Annamanedi and Kalle, 2014). However, the antibacterial mechanism of action of celecoxib and its potential clinical application remain underexplored.

In this study, we investigated the antibacterial activity of celecoxib, as well as the spectrum of its activity against various clinical isolates of multidrug-resistant Gram-positive and Gram-negative pathogens. We also investigated its mechanism of action and validated its *in vivo* antimicrobial efficacy in two different animal models, including *Caenorhabditis elegans* and mouse models of MRSA infection. Additionally, we investigated the immunomodulatory activity of celecoxib in a topical application against MRSA skin infection. Finally, we tested the activity of celecoxib in combination with various antimicrobial agents to investigate the potential for synergistic activities.

## Materials and Methods

### Bacterial Strains and Reagents

The bacterial strains used in this study are presented in **Tables 1–3**. Müller–Hinton Broth (MHB) was purchased from Sigma–Aldrich. Trypticase soy broth (TSB), trypticase soy agar (TSA), and mannitol salt agar (MSA) were purchased from Becton, Dickinson (Cockeysville, MD, USA). Celecoxib was purchased from TSZ chemicals. Vancomycin hydrochloride was obtained from Gold Biotechnology; linezolid from Selleck Chemicals, mupirocin from Aapplichem, NE, clindamycin from TCI Chemicals, and fusidic acid and rifampicin from Sigma–Aldrich.

**Table 1:** Minimum inhibitory concentration (MIC) of celecoxib against Gram-positive bacteria.

Bacteria	Description	Celecoxib (μg/ml)
Methicillin-resistant *Staphylococcus aureus* ATCC 4330	Clinical isolate resistant to methicillin and oxacillin	32
Vancomycin-resistant *S. aureus* (VRSA10)	Resistant to ciprofloxacin, clindamycin, erythromycin, and gentamicin	32
*Streptococcus pneumoniae* ATCC 49619	Isolated from sputum of 75-year-old male, Phoenix, AZ, USA	64
*Bacillus anthracis*	Stern vaccine strain	16
*B. anthracis* UM23	Weybridge strain which contains the toxigenic pXO1 plasmid and lacks the pXO2 capsule plasmid	16
*B. anthracis* AMES35	Isolated from 14-month-old heifer that died in Texas in 1981. It is a derivative of *B. anthracis*, strain Ames that was treated with novobiocin to cure it of the pXO2 plasmid.	16
*B. subtilis* CU 1065	-	16
*Listeria monocytogenes*	F4244 CDC. Clinical isolate from patient cerebrospinal fluid (CSF)	32
*Mycobacterium smegmatis* ATCC 14468	Reference strain	16

**Table 2:** Minimum inhibitory concentration of celecoxib against Gram-negative bacteria.

Bacteria	Description	MIC of celecoxib (μg/ml)		
		(-)	(+) Sub-inhibitory concentration of colistin	(+) Sub-inhibitory concentration of reserpine
*Pseudomonas aeruginosa* ATCC15442	Isolated from animal room water bottle	>256	16	>256
*P. aeruginosa* ATCC BAA-1744	Clinical isolate and VITEK 2 GN identification card quality control organism	>256	16	>256
*Escherichia coli* O157:H7ATCC 700728	Non-toxigenic and quality control strain	>256	16	>256
*Acinetobacter baumannii* ATCC BAA1605	MDR strain isolated from the sputum of a Canadian soldier	>256	8	>256
*A. baumannii* ATCC BAA747	Human clinical specimen -ear pus	>256	16	>256
*Salmonella Typhimurium* ATCC 700720	Wild type strain isolated from a natural source	>256	32	>256
*Klebsiella pneumoniae* ATCC BAA 2146	Clinical isolate New Delhi Metallo-β-Lactamase (NDM-1)	>256	8	>256
*K. pneumoniae* ATCC BAA 1705	Clinical isolate with Carbapenemase (KPC) resistant to carbapenem	>256	16	>256
*E. coli* 1411	Wild type strain	>256	ND	ND
*E. coli* SM1411 Δ *acrAB*	Mutant for *acrAB* efflux pump	64	ND	ND

**Table 3:** Minimum inhibitory concentration of celecoxib against clinical isolates of *S. aureus* strains.

Strain type	Strain ID	Source	Phenotypic properties	Celecoxib (μg/ml)
Methicillin resistant	USA100	U. S. (OH)	Resistant to ciprofloxacin, clindamycin,	32
*S. aureus* (MRSA)			erythromycin	
	USA200	U. S. (NC)	Resistant to clindamycin, methicillin	32
			erythromycin, gentamicin,	
	USA300	U. S. (MS)	Resistant to erythromycin, methicillin, tetracycline	32
	USA400	U. S. (ND)	Resistant to methicillin, tetracycline	16
	USA500	U. S. (CT)	Resistant to ciprofloxacin, clindamycin,	32
			erythromycin, gentamicin,	
			methicillin, tetracycline, trimethoprim	
	USA700	U. S. (LA)	Resistant to erythromycin, methicillin	32
	USA800	U. S. (WA)	Resistant to methicillin	32
	USA1000	U. S. (VT)	Resistant to erythromycin, methicillin	32
	USA1100	U. S. (AL)	Resistant to methicillin	32
	NRS194	U. S. (ND)	Resistant to methicillin	32
	NRS108	France	Resistant to gentamicin	32
	NRS119	U. S. (MA)	Resistant to linezolid	16
	ATCC 43300	U. S. (KS)	Resistant to methicillin	32
	ATCC BAA-44	Lisbon, Portugal	Multidrug-resistant strain	32
	NRS70	Japan	Resistant to erythromycin, clindamycin, spectinomycin	32
	NRS71	UK	Resistant to tetracycline, methicillin	32
	NRS100	U. S.	Resistant to tetracycline, methicillin	32
	NRS107	U. S.	Resistant to methicillin, mupirocin	32
Vancomycin-intermediate	NRS1	Japan	Resistant to aminoglycosides and	32
*S. aureus* (VISA)			tetracycline; glycopeptide- intermediate *S. aureus*	
	NRS19	U. S. (IL)	Glycopeptide-intermediate *S. aureus*	32
	NRS37	France	Glycopeptide-intermediate *S. aureus*	32
Vancomycin-resistant	VRS1	U. S.	Resistant to vancomycin	128
*S. aureus* (VRSA)	VRS2	U. S.	Resistant to vancomycin, erythromycin, spectinomycin	128
	VRS3a	U. S.	Resistant to vancomycin	32
	VRS3b	U. S.	Resistant to vancomycin	32
	VRS4	U. S.	Resistant to vancomycin, erythromycin, spectinomycin	128
	VRS5	U. S.	Resistant to vancomycin	16
	VRS6	U. S.	Resistant to vancomycin	16
	VRS7	U. S.	Resistant to vancomycin, β-lactams	128
	VRS8	U. S.	Resistant to vancomycin	32
	VRS9	U. S.	Resistant to vancomycin	64
	VRS11a	U. S.	Resistant to vancomycin	32
	VRS11b	U. S.	Resistant to vancomycin	32
	VRS12	U. S.	Resistant to vancomycin	32
	VRS13	U. S.	Resistant to vancomycin	32

### Antibacterial Assays

Minimum inhibitory concentrations (MICs) were determined in triplicate, in Mueller–Hinton broth, using the broth micro dilution method described by the Clinical and Laboratory Standards Institute (CLSI; Mohamed et al., 2014). The MIC was interpreted as the lowest concentration of the drug able to completely inhibit the visible growth of bacteria after incubating plates for at least 16 h at 37°C. The highest MIC value taken from two independent experiments was reported.

### Gram-Negative Outer Membrane Permeability Assay

The MIC of celecoxib in the presence of colistin was measured as described in the antibacterial assays section, above. Sub-inhibitory concentration of colistin (0.065–0.25 μg/ml, **Table 2**) was added to the media to increase outer membrane permeability and facilitate the entrance of celecoxib. The following sub-inhibitory concentration of colistin was used; *P. aeruginosa* ATCC15442 and *S almonella Typhimurium* (0.25 μg/ml), *P. aeruginosa* ATCC BAA-1744 and *Klebsiella pneumoniae* (0.125 μg/ml), *Escherichia coli* O157:H7ATCC 700728 and *Acinetobacter baumannii* (0.0625 μg/ml).

### Effect of Efflux Pump on Celecoxib Activity

The effect of efflux pumps on the ability of celecoxib to gain entry into Gram-negative bacteria was investigated using known efflux pump inhibitor (reserpine) and efflux pump deletion mutant strain of *E. coli*. The MIC of celecoxib was examined in the presence of sub-inhibitory concentration of reserpine (32 μg/ml) against all strains of Gram-negative bacteria used in this study. Efflux pump deletion mutant *E. coli* SM1411 Δ *acrAB* was employed to determine if *acrAB* efflux pump plays a role in contributing to intrinsic resistance to celecoxib as described before (O’Neill et al., 2002; Randall et al., 2013).

### Time Kill Assay

The time kill assay was performed as described before (Mohamed et al., 2014). Briefly, MRSA USA300 was diluted to 1 × 10^6^ CFU/mL and treated with 4X MIC of control antimicrobials (vancomycin or linezolid), 4X and 8X MIC of celecoxib (in triplicates) in MHB. Cultures were incubated at 37°C and samples were collected at indicated time points to count MRSA colony forming units (CFU).

### Macromolecular Synthesis Assay

*Staphylococcus aureus* strain ATCC 29213 was grown overnight on TSA plates and the isolated colonies were cultured in 15 ml of MHB to an early exponential phase (OD_600_ = 0.2–0.3). Aliquots (100 μl) of the early exponential phase culture were added to triplicate wells of a 96-well microtiter plate. Antibiotics with known mechanisms of action (ciprofloxacin, rifampicin, linezolid, vancomycin, and cerulenin) and auranofin were added to the plate as controls. DMSO was added to the control groups. After 30 min of incubation at 37°C, radiolabeled precursors such as [3H] thymidine (0.5 μCi), [3H] uridine (0.5 μCi), [3H] leucine (1.0 μCi), [14C] *N*-acetylglucosamine (0.4 μCi), and [3H] glycerol (0.5 μCi) were added to quantify the amount of for DNA, RNA, protein, cell wall, and lipid synthesis, respectively. Reactions measuring the inhibition of DNA and RNA synthesis were stopped after 15 min by the addition of 5% trichloroacetic acid (TCA). Then, the tubes were chilled on ice for 30 min. The TCA-precipitated materials were collected on a 25 mm GF/1.2 μM PES 96-well filter plate. Filters were washed five times with 5% TCA, dried, and then counted using a Packard Top Count microplate scintillation counter. Reaction wells measuring the inhibition of protein synthesis were stopped after 40 min, precipitated, and counted in a manner similar to that used for the DNA and RNA synthesis inhibition assays. Reaction wells measuring the inhibition of cell wall synthesis were stopped after 40 min by the addition of 8% SDS and then heated for 30 min at 95°C. After cooling, the material were spotted onto nitrocellulose membrane filters (0.8 μM) and washed three times with 0.1% SDS. Filters were dried and counted using a Beckman LS3801 liquid scintillation counter. Reactions measuring the inhibition of lipid synthesis were stopped after 40 min by the addition of chloroform/methanol (1:2) and centrifuged at 13,000 rpm for 10 min. Then, the organic phase was carefully transferred to a scintillation vial, dried, and counted using liquid scintillation counting. Incorporation of radiolabeled DNA, RNA, protein, cell wall, and lipid precursors was quantified using the scintillation data and inhibition was calculated. Results were presented as the percent inhibition of each macromolecular synthesis pathway.

### Toxicity Assay in *C. elegans*

*Caenorhabditis elegans* AU37 (sek-1; glp-4) strain glp-4(bn2) were used for the toxicity studies. L4-stage worms were synchronized as described previously (Alajlouni and Seleem, 2013). Synchronized worms (∼20 worms) in 50% M9 buffer and 50% TSB were added to each well of a 96-well plate. Drugs (celecoxib and linezolid) at indicated concentrations (16 or 32 μg/ml) were added to the wells and the plates were incubated for 4 days at room temperature. Worms were assessed every day; the percentage of worms remaining alive in each group was calculated.

### Efficacy of Celecoxib in MRSA-Infected *C. elegans*

*Caenorhabditis elegans* AU37 (sek-1; glp-4) strain glp-4(bn2) was used to test the *in vivo* antimicrobial efficacy of celecoxib as described previously (Alajlouni and Seleem, 2013). *S. aureus* strain MRSA USA300 was used for infection and the MIC of control antibiotic (linezolid) and celecoxib against MRSA USA300 were 2 and 32 μg/ml, respectively. Briefly, L4-stage worms were infected with MRSA USA300 for 8 h at room temperature. The worms were washed with M9 buffer, and then drugs (celecoxib and linezolid) at indicated concentrations were added to the 96-well plates containing approximately 20 worms per well. After 24 h, the worms were washed four times with PBS and 100 mg of sterile, 1.0-mm silicon carbide particles (Biospec Products, Bartlesville, OK, USA) were added to each tube. Worms were disrupted by vortexing the tubes at maximum speed for 1 min. The final suspension containing MRSA was plated onto MSA plates to count the bacteria. The total CFU count in each well was divided by the number of worms present in the respective well. The results shown are the percent reduction in CFU per worm, compared with an untreated control.

### Efficacy of Celecoxib in MRSA-Infected *Mice*

Eight-week-old female BALB/c mice (Harlan Laboratories, Indianapolis, IN, USA) were used in this study. All animal procedures were approved by the Purdue University Animal Care and Use Committee (PACUC). The mouse model of MRSA skin infection was performed as described previously (Cho et al., 2010, 2011; Mohamed and Seleem, 2014). Briefly, mice were infected intradermally with 1.65 × 10^8^ CFU MRSA300. After 48 h of infection, open wounds formed and the mice were divided into five groups of five mice each. Two groups were treated topically with 20 mg of either 1 or 2% celecoxib in petroleum jelly. One group received the vehicles alone (20 mg petroleum jelly). Another group was treated topically with 20 mg of 2% fusidic acid in petroleum jelly and the last group was treated orally with clindamycin (25 mg/kg). All groups were treated twice a day for 5 days. 24 h after the last treatment, the skin area around the wound was swabbed with 70% ethanol and the wound (around 1 cm^2^) was precisely excised and homogenized. Bacteria in the homogenate were counted using MSA plates.

### Determination of Cytokine Levels

Skin homogenates obtained from infected mice were centrifuged at 4000 rpm for 10 min and the supernatants were used for the detection of cytokine levels. Tumor necrosis factor-α (TNF-α), interleukin-6 (IL-6), interleukin-1 beta (IL-1β), and monocyte chemo attractant protein-1 (MCP-1) ELISA kits (R&D Systems, Inc.) were used to determine the levels of these cytokines according to the manufacture’s instruction (Rioja et al., 2004).

### Synergy Assay

Synergy between celecoxib and conventional antimicrobials (gentamicin, clindamycin, vancomycin, linezolid, daptomycin, retapamulin, fusidic acid, and mupirocin) in the treatment of four clinical isolates of *S. aureus* (MRSA300, NRS107, NRS119, and VRSA5) was evaluated using the Bliss Independence Model, as described previously (Morones-Ramirez et al., 2013). Synergy (S) was calculated using the formula: *S = (f_A0_/f_00_)(f_0B_/f_00_) – (f_AB_/f_00_).* The parameter *f_AB_* refers to the optical density of the bacteria grown in the presence of celecoxib and antibiotics; parameters *f_A0_* and *f_0B_* refer to the bacterial growth rate in the presence of antibiotics alone and celecoxib alone, respectively; the parameter *f_00_* refers to the bacterial growth in the absence of drugs. Degree of synergy (*S)* values corresponds to the following cut-offs: Zero indicates neutral, values above zero (positive value) represents synergism, and values below zero (negative values) correspond to antagonism. Drug combinations with higher positive value represents high degree of synergism.

### Statistical Analyses

Statistical analyses were performed using GraphPad Prism 6.0 software (GraphPad Software, La Jolla, CA, USA). *P*-values were calculated by using two-tailed unpaired Student *t*-tests. *P-*values <0.05 were considered significant.

## Results

### Antibacterial Activity

The antibacterial activity of celecoxib was tested using various important multidrug-resistant strains of Gram-positive (**Table 1**) and Gram-negative (**Table 2**) pathogens. Celecoxib showed activity against all Gram-positive bacteria tested, including methicillin- and vancomycin-resistant *S. aureus* (VRSA), *Streptococcus pneumonia, Listeria monocytogenes, Bacillus anthracis, B. subtilis*, and *M. smegmatis*, with MICs ranging from 16 to 64 μg/ml (**Table 1**). In contrast, celecoxib alone did not show antibacterial activity against Gram-negative bacteria. However, when the outer membranes of Gram-negative bacteria were compromised with a sub-inhibitory concentration of colistin, celecoxib showed antimicrobial activity against all Gram-negative pathogens tested, including *P. aeruginosa, E. coli, K. pneumonia, S. Typhimurium, A. baumannii*, with MICs ranging from 8 to 32 μg/ml (**Table 2**). In addition, there was a fourfold decrease in celecoxib’s MIC observed in an acrAB mutant *E. coli* as compared to the wild type strain. We did not observe any change in the MIC with addition of the efflux pump inhibitors reserpine (**Table 2**).

The antibacterial activity of celecoxib was also assessed using a series of multidrug-resistant *S. aureus* clinical isolates (**Table 3**). The MIC of celecoxib required to inhibit 90% (MIC_90_) of the MRSA and vancomycin-intermediate *S. aureus* (VISA) clinical isolates was found to be 32 μg/ml. However, the MIC_90_ of celecoxib against VRSA clinical isolates tested was 128 μg/ml.

### Killing Kinetics of *S. aureus* by Celecoxib

**FIGURE 1:**
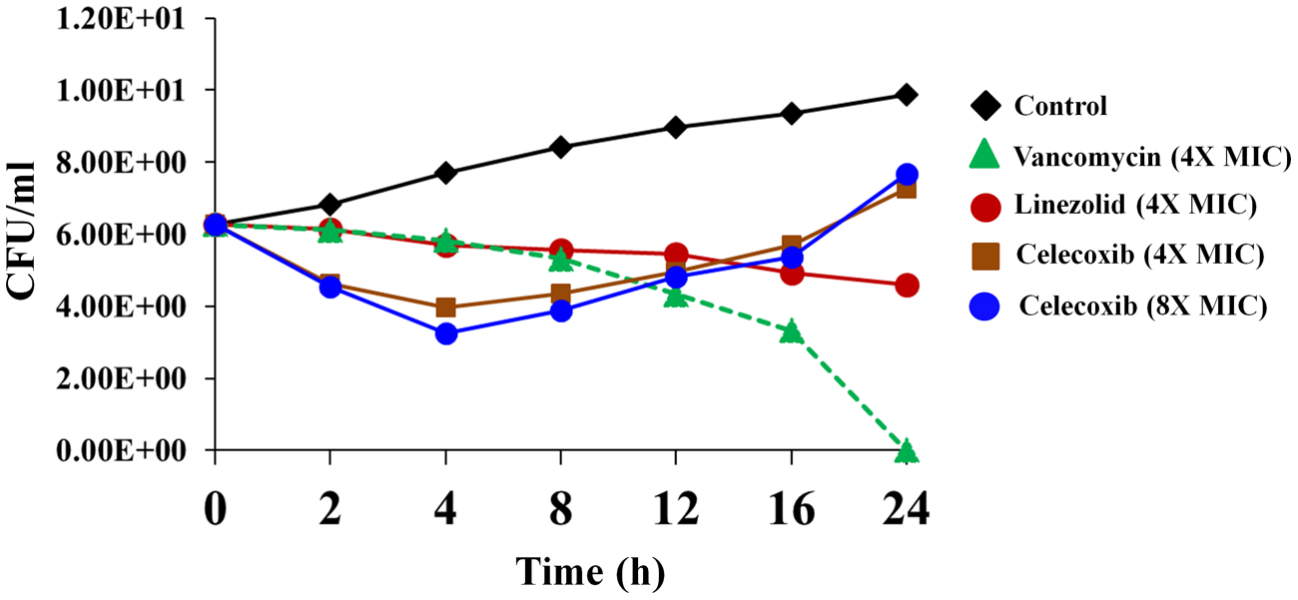
**Time-kill assay for celecoxib tested against *Staphylococcus aureus*.** Killing kinetics of celecoxib (4X and 8X MIC), vancomycin (4X MIC), and linezolid (4X MIC), against methicillin-resistant *S. aureus* (MRSA) USA300 in MHB are shown. The results are presented as means ± SD (*n* = 3). Data without error bars indicate that the SD is too small to be seen.

We investigated the rate of bacterial killing by celecoxib. As seen in **Figure 1**, MRSA USA300 treated with 4X and 8X MIC of celecoxib exhibits a biphasic killing pattern. Treatment with celecoxib consists of an initial rapid bactericidal phase (2.49 ± 0.23 log_10_ and 3.01 ± 0.26 log_10_ CFU reduction at 4 h with 4X and 8X MIC) followed by a regrowth of MRSA. In comparison, vancomycin had a bactericidal activity after 24 h, while linezolid treatment results in single log reduction after 24 h incubation exhibiting a bacteriostatic activity.

### Mechanism of Action

**FIGURE 2:**
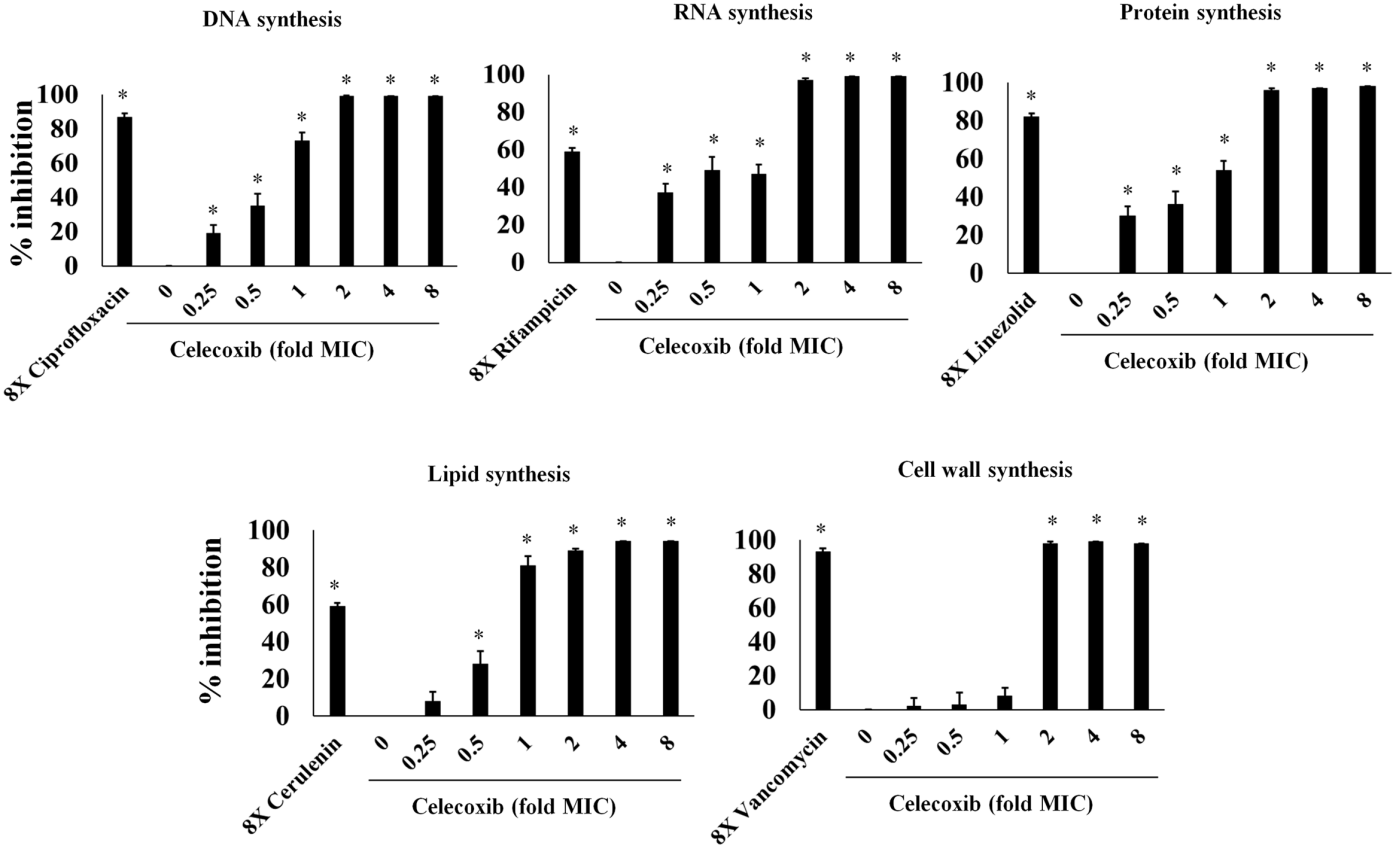
**Macromolecular synthesis assay in the presence of celecoxib and control antibiotics.** Incorporation of radiolabeled precursors such as [3H] thymidine, [3H] uridine, [3H] leucine, [14C] *N*-acetylglucosamine and [3H] glycerol for DNA, RNA, protein, cell wall, and lipid synthesis, respectively, were quantified in *S. aureus* ATCC 29213. Based on the incorporation of radiolabeled precursors, percent of inhibition by celecoxib at concentration dependent manner was examined. Control antibiotics including ciprofloxacin (DNA), rifampicin (RNA), linezolid (protein), cerulenin (lipid synthesis), and vancomycin (cell wall synthesis) at 8X MIC were used. Triplicate samples were used for each group and the statistical analysis was calculated by the two-tailed Student *t*-test. All treatment groups were compared to untreated control group. *P*-value of (^∗^*P* ≤ 0.05) is considered as significant.

In view of the results demonstrating broad-spectrum antibacterial activity, we used macromolecular synthesis assays in *S. aureus* ATCC 29213 to investigate the antibacterial mode of action of celecoxib. As shown in **Figure 2**, RNA, DNA, and protein synthesis inhibition were detected at concentrations significantly below the MIC (0.25X). However, a secondary effect was also observed at higher concentration, with a clear dose-dependent disruption of [3H] glycerol incorporation indicating decreased lipid synthesis. Cell wall synthesis inhibition was evident only at a concentration above the MIC (2X).

### Toxicity in *C. elegans*

**FIGURE 3:**
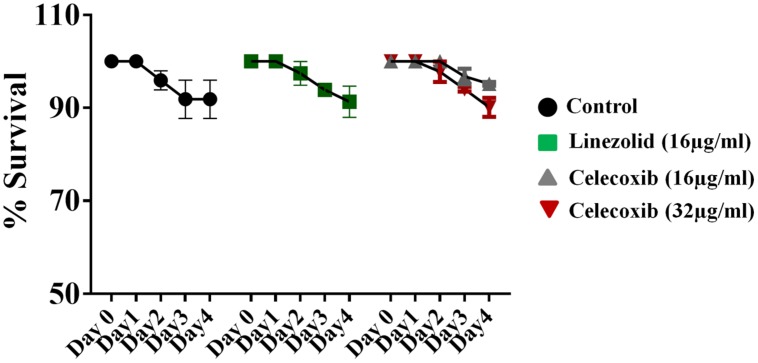
**Evaluation of toxicity in *Caenorhabditis elegans* model.** *C. elegans* strain glp-4; sek-1 (L4-stage) were grown for four days in the presence of celecoxib (16 and 32 μg/ml) and linezolid (16 μg/ml). Worms were monitored daily and the live worms were counted. Results were expressed as percent live worms in relative to the untreated control groups. Triplicate wells were used for each group and the results were means ± SD (*n* = 3).

The safety of celecoxib was evaluated in a *C. elegans* whole-animal model. As shown in **Figure 3**, *C. elegans* treated with 16 or 32 μg/ml of celecoxib for 4 days did not show any significant toxicity. These results are similar to those seen in the linezolid (16 μg/ml) and untreated control groups.

### Efficacy in Animal Models

**FIGURE 4:**
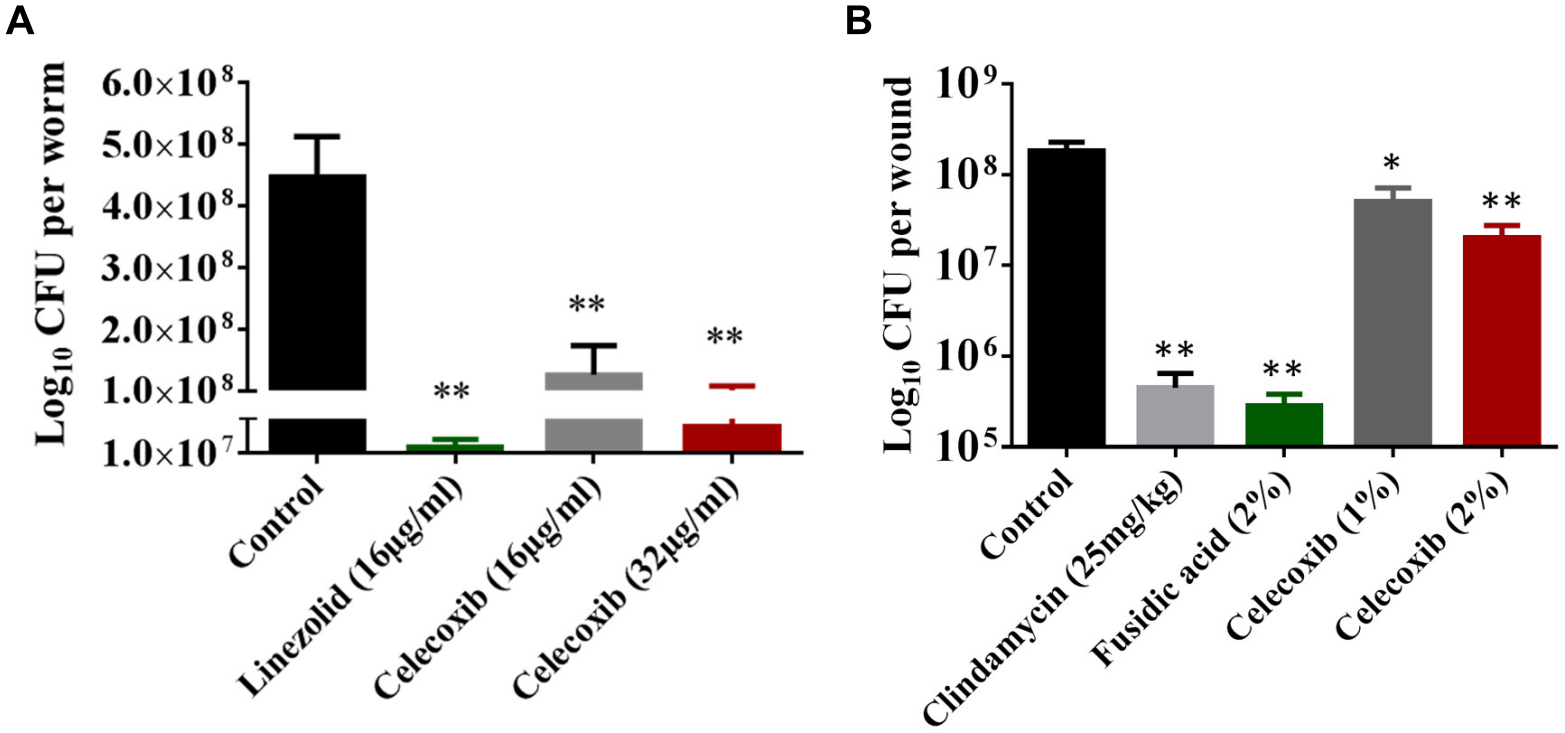
**Efficacy of celecoxib in MRSA-infected animal models. (A)** L4-stage worms infected with MRSA USA300 were treated with celecoxib (16 and 32 μg/ml) and linezolid (16 μg/ml) for 24 h. At this point, the worms were disrupted and the amount of MRSA in the lysate (CFU) was determined. CFU per worm in treated groups relative to the untreated control groups were shown. Triplicate wells were used for each group and the results were means ± SD (*n* = 3). **(B)** Efficacy of treatment of MRSA-infected mouse skin lesions with celecoxib 1 and 2%, clindamycin (25 mg/kg), fusidic acid 2%, and petroleum jelly (negative control) twice daily for 5 days were evaluated. Five mice per group was used and the results were means ± SD of five mice. CFU per wound was calculated and presented. ^∗^*P* ≤ 0.05 and ^∗∗^*P* ≤ 0.01 were considered as significant.

Having demonstrated a comfortable safety profile, the antibacterial efficacy of celecoxib was tested in a *C. elegans*, whole-animal MRSA infection model. As seen in **Figure 4A**, celecoxib treatment significantly reduced the mean bacterial count, compared with the untreated control. Treatment with celecoxib at 16 and 32 μg/ml significantly decreased the bacterial CFU of 0.56 ± 0.33 log_10_ and 0.94 ± 0.43 log_10_, respectively. For comparison, linezolid at 16 μg/ml had significant reduction in bacterial CFU (0.99 ± 0.17 log_10_), compared with the untreated control.

Next we tested the *in vivo* antibacterial efficacy of celecoxib in a mouse model of MRSA skin infection. As shown in **Figure 4B**, all treatment groups (1 or 2% celecoxib, 2% fusidic acid, or clindamycin oral treatment) significantly reduced the mean bacterial counts, compared with the control group (*P* ≤ 0.05). Groups treated topically with 1 and 2% celecoxib had a reduction in MRSA CFU of 0.66 ± 0.19 log_10_ and 1.02 ± 0.27 log_10_, respectively. Topical treatment with 2% fusidic acid and oral clindamycin (25 mg/kg) treatment reduced the bacterial load of 2.90 ± 0.23 log_10_ and 2.40 ± 0.32 log_10_ CFU, respectively.

### Effect of Celecoxib on Inflammatory Cytokine Levels Induced by MRSA Skin Infection

**FIGURE 5:**
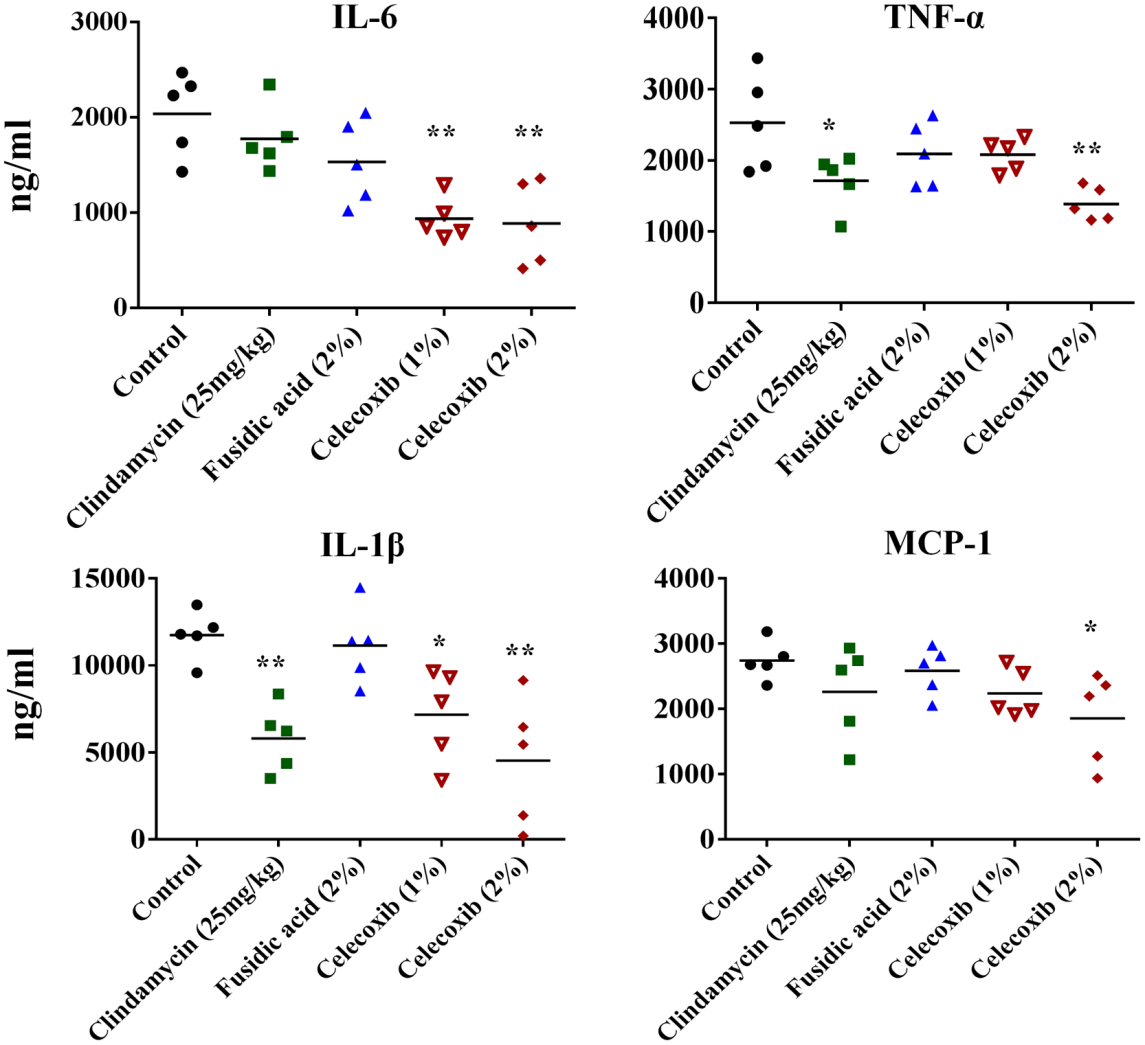
**Effect of celecoxib on IL-6, TNF-α, IL-1β, and MCP-1 production in MRSA infected skin lesions.** Supernatants from skin homogenates were used for cytokine detection by ELISA. Each point represents single mice and each group has five mice. Statistical analysis was calculated by the two-tailed Student’s *t*-test. *P*-values of ^∗^*P* ≤ 0.05, ^∗∗^*P* ≤ 0.01 are considered as significant.

We investigated the immune-modulatory activity of celecoxib in MRSA skin infection by measuring the levels of the inflammatory cytokines IL-6, TNF-α, IL-1β, and MCP-1 using ELISA. As shown in **Figure 5**, treatment with 2% celecoxib significantly reduced the levels of all tested inflammatory cytokines, compared with an untreated control. Treatment with 1% celecoxib significantly reduced the levels of IL-6 and IL-1β. Clindamycin treatment also reduced levels of TNF-α and IL-1β.

### Synergism with Topical and Systemic Antimicrobials

**FIGURE 6:**
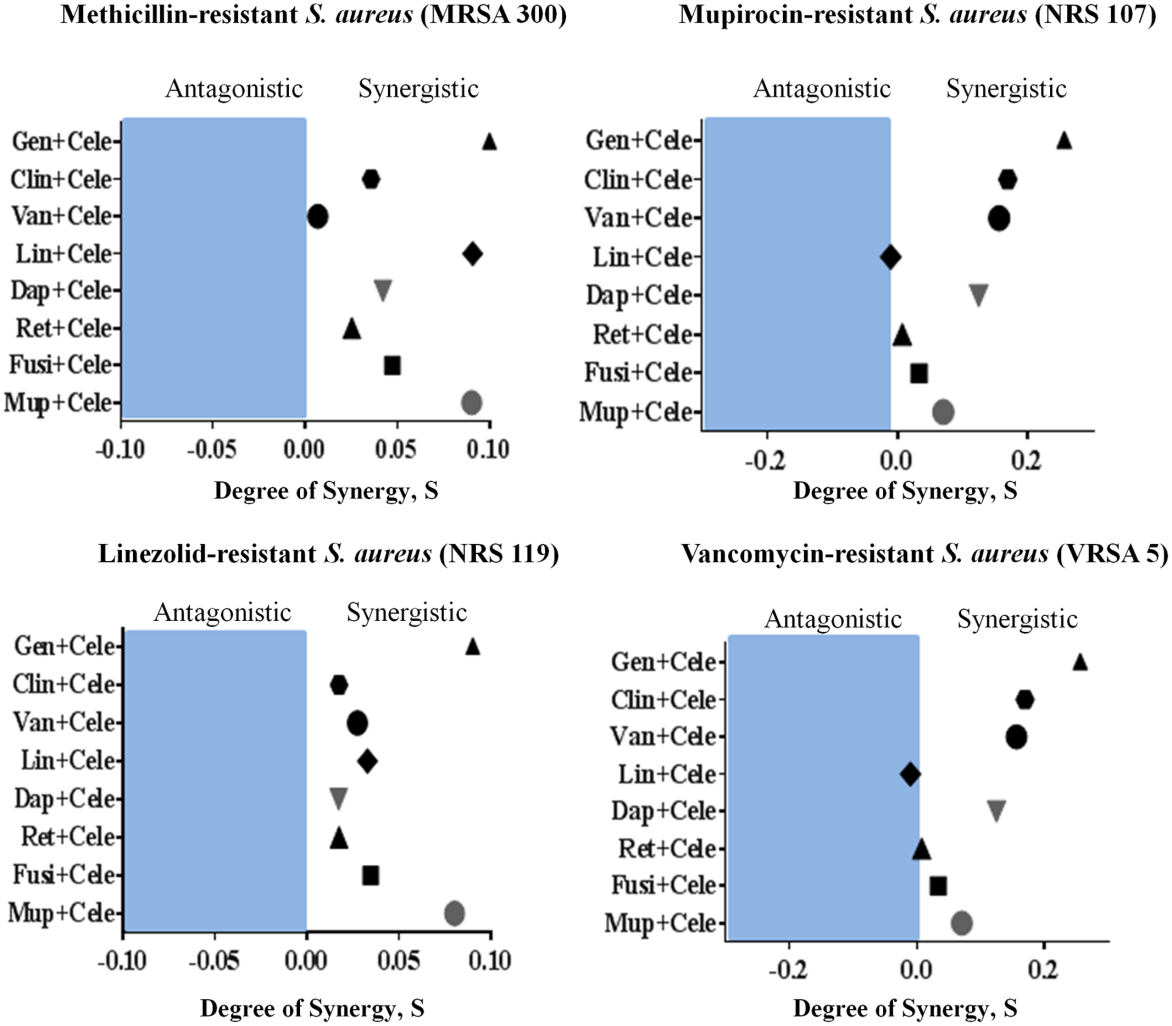
**Synergistic activity of celecoxib with topical and systemic antimicrobials.** The Bliss Independence Model confirms a synergistic effect between celecoxib and conventional antimicrobials against various drug-resistant strains of *S. aureus* (MRSA300, NRS119, NRS107, and VRSA5). The degree of synergy was quantified after 12 h of treatment with celecoxib (8 μg/ml) in combination with sub-inhibitory concentrations of topical (mupirocin, fusidic acid, daptomycin, and retapamulin) and systemic antimicrobials (gentamicin, clindamycin, vancomycin, and linezolid).

The antimicrobial activities of combinations of celecoxib with topical and systemic antimicrobials were investigated *in vitro*, using the Bliss independence model, with clinical isolates of multidrug-resistant *S. aureus*. Celecoxib acted synergistically with all tested antimicrobials (with the exception of linezolid) against all strains of multi-drug resistant *S. aureus* tested, including MRSA300, VRSA5, linezolid-resistant *S. aureus* (NRS119), and mupirocin-resistant *S. aureus* (NRS107). However, celecoxib showed slight antagonism when combined with linezolid against VRSA5 (**Figure 6**).

## Discussion

The emergence of bacterial resistance is not a new phenomenon. However, because only a few antibiotics have been developed over the past few decades, the continuous evolution and spread of multidrug-resistant bacterial strains is a serious threat to the public health (Centers for Disease Control and Prevention [CDC], 2013). The pharmaceutical companies’ lack of interest in antimicrobial research and development has also become a major concern (Thangamani et al., 2015). The World Health Organization has already warned that we are heading toward a “post-antibiotic era” and suggested that urgent measures need to be taken (Aryee and Price, 2015). Therefore, recent research had been directed toward finding new antimicrobials and novel strategies to combat multidrug-resistant bacterial pathogens. One promising approach gaining increased attention is the repurposing of existing approved drugs as antimicrobials.

In an attempt to repurpose approved drugs, we and others (Chiu et al., 2009, 2012; Pereira et al., 2013) have found that celecoxib exhibits broad-spectrum antimicrobial activity against Gram-positive and Gram-negative bacterial pathogens. Celecoxib, a classical Non-steroidal anti-inflammatory drug (NSAID) and inhibitor of the enzyme COX2, has been widely used as an anti-inflammatory drug for the treatment of acute pain, arthritis, menstrual pain, and discomfort (Tindall, 1999; Bensen, 2000; Kumar et al., 2013). Independent of its anti-inflammatory action, celecoxib exhibits antimicrobial activity against *F. tularensis* and *S. aureus* (Chiu et al., 2009, 2012). Celecoxib also reduces *H. capsulatum* burden by enhancing phagocytosis of alveolar macrophages and decreasing levels of inflammatory cells and cytokines, thereby exhibiting a protective role in pathogenesis of *H. capsulatum* (Pereira et al., 2013). Our study demonstrated that celecoxib possesses activity against various multidrug-resistant Gram-positive bacteria, including *S. aureus, S. pneumonia, L. monocytogenes, B. anthracis, B. subtilis*, and *M. smegmatis*. However, we noticed that Gram-negative pathogens are not susceptible to celecoxib, and the lack of activity was found to be due to the permeability barrier conferred by the outer membrane. This was further confirmed by the fact that the antimicrobial activity of celecoxib against Gram-negative bacteria was restored when the integrity of the outer membrane was compromised using a sub-inhibitory concentration of colistin (Vaara, 2010; Vaara et al., 2010; Velkov et al., 2013). In addition, celecoxib also showed activity when an efflux pump such as *acrAB* was deleted in *E. coli.* AcrAB has been known to contribute for resistant phenotype for various antibiotics including ampicillin, chloramphenicol, and rifampicin (Okusu et al., 1996). Taken together, in addition to the intrinsic physical barrier outer membrane, celecoxib entry into Gram-negative bacteria is also influenced by efflux pumps such as AcrAB. Our results indicate that the target of celecoxib is present in both Gram-positive and Gram-negative bacteria and that celecoxib can be combined with other approved drugs that cause leakage in the outer membrane, such as colistin, to sensitize Gram-negative pathogens. Next, we investigated the activity of celecoxib against clinical isolates of multidrug-resistant *S. aureus*. Celecoxib inhibited the growth of all tested clinical isolates of MRSA, VISA, VRSA, linezolid-resistant *S. aureus* (NRS119), and mupirocin-resistant *S. aureus* (NRS107). MIC values determined in our study for celecoxib against MRSA correlates with MIC values reported for celecoxib against *F. tularensis* and *S. aureus* in previous published studies (Chiu et al., 2009, 2012).

Time kill kinetics of celecoxib against *S. aureus* revealed a unique biphasic killing pattern. The bactericidal effect of celecoxib lasted for only a short time, after which gradual regrowth of bacteria was noticed. This pattern of inhibition and regrowth was reported for some antibiotics such as azlocillin and tobramycin against *P. aeruginosa* (White et al., 1980; McFarland et al., 1994). We suspected that the regrowth might be due to resistant bacterial subpopulation but our attempts to isolate a stable mutants to celecoxib failed. Additionally, at high MIC, celecoxib tends to precipitate in the growth media which might attributed to the regrowth of bacteria.

The mechanism of celecoxib’s broad-spectrum antibacterial activity remains unidentified. In our study, we found that celecoxib inhibited the synthesis of DNA, RNA, and protein at concentrations significantly below the MIC. Additionally, the disruption of lipid synthesis was evident at higher MIC concentration, whereas no significant effect was observed on the cell wall synthesis. These results indicate that perturbation of the lipid synthesis by celecoxib might be a secondary effect due to the early RNA and protein synthesis inhibition. The effect of celecoxib on multiple macromolecular synthesis pathways indicating that celecoxib may in fact have a complex mode of action that involves inhibition of multiple targets in *S. aureus* or it may disrupt general cellular energy metabolism. Further, we attempted to generate a *S. aureus* mutant that is resistant to celecoxib. No colonies resistant to celecoxib at three-, five-, or tenfold the MIC were detected. In addition, serial passage of *S. aureus* with sub-inhibitory concentration of celecoxib for 12 days did not result in mutants resistant to celecoxib. The potential inhibition of multiple bacterial enzymes and pathways by celecoxib may help explain our inability to isolate spontaneous celecoxib-resistant mutants. Future studies are warranted to identify the precise molecular target (s) of celecoxib.

In view of the broad spectrum antibacterial activity exhibited by celecoxib *in vitro*, we decided to investigate the *in vivo* antibacterial activity of celecoxib in animal models of MRSA infection. First we tested the efficacy in MRSA infected *C. elegans.* Whole animal model including *C. elegans*, provides a great platform for validating the *in vivo* efficacy of novel compounds (Alajlouni and Seleem, 2013; Rajamuthiah et al., 2015). In addition, *C. elegans* models enables simultaneous assessment of efficacy and toxicity of the tested drugs, reduces the associated cost of drug discovery and lowers the burden for extensive animal testing (Alajlouni and Seleem, 2013; Rajamuthiah et al., 2015). Our results indicates that celecoxib at 16 and 32 μg/ml, which are concentrations without considerable toxicity to the host, significantly reduced the mean bacterial load (by 71 and 85%, respectively) when compared with a control group (*P* ≤ 0.05). Celecoxib at 32 μg/ml had an effect on the mean bacterial count that was comparable to that of linezolid (16 μg/ml). Next, we moved forward to validate celecoxib’s efficacy in a mouse model of MRSA infection. However, a high MIC that cannot be achieved systemically is a major impediment to the potential use of celecoxib as an antimicrobial agent. While the use of celecoxib to treat systemic bacterial infections is not currently possible, local application of celecoxib for treating/preventing bacterial infections in wounds is a novel application for this drug that holds considerable promise. Therefore we decided to test the activity of celecoxib in a topical MRSA skin infection model. Celecoxib 1 and 2% significantly reduced the bacterial load in the wounds (by 72 and 87%, respectively) when compared with a control group (*P* ≤ 0.05).

However, staphylococcal skin infections and exotoxins secreted by *S. aureus* often induce excess host inflammatory cytokines, which in turn aggravate the pathogenesis of the disease (Montgomery et al., 2013; Sharma-Kuinkel et al., 2013).This aggravated inflammatory cascade is thought to play a greater role in the severity of *S. aureus* skin infections more than the size of the bacterial burden (Montgomery et al., 2013; Sharma-Kuinkel et al., 2013). Additionally, inflammation has been shown to delay healing and to result in increased scarring (Eming et al., 2007). Drugs with anti-inflammatory properties, especially those that inhibit pro-inflammatory cytokines such as IL-6 and TNF-α, would accelerate the healing of chronic wounds. (Tindall, 1999; Fournier and Philpott, 2005; McCormack, 2011; Kumar et al., 2013; Sharma-Kuinkel et al., 2013). Celecoxib, which is known to have anti-inflammatory activity, would potentially be able to limit the inflammatory process induced by MRSA infection. Therefore, we measured the inflammatory cytokines in MRSA lesions treated with celecoxib. Topical treatment with celecoxib 1% significantly (*P* ≤ 0.05) reduced levels of TNF-α and IL-1β, while celecoxib 2% significantly (*P* ≤ 0.05) reduced the levels of all the inflammatory cytokines measured (IL-6, TNF-α, IL-1β, and MCP-1). This ability of celecoxib to dampen the inflammatory response might aid the healing of chronic wounds (Wallace and Stacey, 1998; Fournier and Philpott, 2005; Cowin et al., 2006; Jialal et al., 2007; Khanna et al., 2010; Donath, 2014). Celecoxib’s recognized beneficial role in the wound healing process, reducing scar formation without disrupting reepithelization, is an added advantage for the treatment of bacterial skin infections (Wilgus et al., 2003).

With increased emergence of resistant strains of *S. aureus* to topical drugs of choice, such as mupirocin and fusidic acid, combination therapies have recently been gaining attention (Farrell et al., 2011; Huang et al., 2011; McNeil et al., 2011; Brynildsen et al., 2013; Hu and Coates, 2013; Mohammad et al., 2015). Identifying other antimicrobial partners capable of being paired with celecoxib can potentially prolong the clinical utility of these antibiotics and reduce the likelihood of emergence of resistant strains. We, therefore, investigated whether celecoxib has potential to be combined with antibiotics against multidrug-resistant *S. aureus* strains by using the Bliss independence model (Morones-Ramirez et al., 2013). Celecoxib was found to exhibit a synergistic relationship with topical (mupirocin, fusidic acid, daptomycin, and retapamulin) and systemic antimicrobials (gentamicin, clindamycin, vancomycin, and linezolid), against most of the tested multidrug-resistant staphylococcal strains, including MRSA300, NRS119, NRS107, and VRSA5. This finding provides a potential basis for the combination of celecoxib with conventional antimicrobial drugs for the treatment staphylococcal skin infections and reducing the likelihood of strains developing resistance to monotherapy. This combination therapy is also expected to overcome some of the limitations associated with celecoxib monotherapy through lowering the required therapeutic dose, though further *in vivo* studies are needed to confirm this point.

Taken together, our results show that celecoxib exhibits several beneficial properties, including broad spectrum antimicrobial activity against various multidrug resistant Gram-positive and Gram-negative pathogens, synergistic action with conventional antimicrobials, and anti-inflammatory activity that reduces excess host inflammation during infection. Celecoxib may, therefore, be a good candidate for repurposing for the treatment of topical bacterial infections. This emerging approach might form a novel alternative strategy in search of new antimicrobials.

## Author Contributions

ST and WY performed animal experiments. MS designed the study. ST and MS analyzed data and wrote the manuscript. All authors discussed the results and commented on the manuscript.

## Conflict of Interest Statement

The authors declare that the research was conducted in the absence of any commercial or financial relationships that could be construed as a potential conflict of interest.
